# The Role of YouTube and the Entertainment Industry in Saving Lives by Educating and Mobilizing the Public to Adopt Behaviors for Community Mitigation of COVID-19: Successive Sampling Design Study

**DOI:** 10.2196/19145

**Published:** 2020-04-21

**Authors:** Charles E Basch, Corey H Basch, Grace C Hillyer, Christie Jaime

**Affiliations:** 1 Teachers College Columbia University New York, NY United States; 2 William Paterson University Wayne, NJ United States; 3 Mailman School of Public Health Columbia University New York, NY United States

**Keywords:** YouTube, COVID-19, social media, pandemic, outbreak, infectious disease, public health, prevention

## Abstract

**Background:**

Effective community mitigation through voluntary behavior change is currently the best way to reduce mortality caused by coronavirus disease (COVID-19). This study builds on our prior study based on the scientific premise that YouTube is one of the most effective ways to communicate and mobilize the public in community mitigation to reduce exposure to severe acute respiratory syndrome coronavirus 2 (SARS-CoV-2).

**Objective:**

Because of the rapidly changing nature of YouTube in the context of the COVID-19 pandemic, we conducted a follow-up study to document how coverage of preventive behaviors for effective community mitigation has changed.

**Methods:**

A successive sampling design was used to compare coverage of behaviors to mitigate community transmission of COVID-19 in the 100 most widely viewed YouTube videos in January 2020 and March 2020.

**Results:**

Videos in the January and March samples were viewed >125 million times and >355 million times, respectively. Fewer than half of the videos in either sample covered any of the prevention behaviors recommended by the US Centers for Disease Control and Prevention, but many covered key prevention behaviors and were very widely viewed. There were no videos uploaded by entertainment television in the January sample, but this source comprised the majority of videos and garnered the majority of cumulative views in the March sample.

**Conclusions:**

This study demonstrates the incredible reach of YouTube and the potential value of partnership with the entertainment industry for communicating and mobilizing the public about community mitigation to reduce mortality from the COVID-19 viral pandemic.

## Introduction

When discussing the goals of community mitigation during a White House press briefing on March 31, 2020, Dr Deborah Birx, the US coronavirus response coordinator, stated that mitigation begins and ends with community [1]. She presented modeling estimates showing that without mitigation, between 1.5 and 2.2 million people in the United States would die from coronavirus disease (COVID-19); however, with effective community mitigation, mortality could be reduced to between 100,000 to 200,000 deaths. These community mitigation efforts recommended by the US Centers for Disease Control and Prevention (CDC) and described in our prior study rely exclusively on voluntary personal behaviors such as staying home, social distancing, and hand hygiene [2]. The scientific premise for that study was that, because of its widespread reach to the American (and global) population, YouTube is one of the most effective ways to communicate with the public and mobilize them to become engaged in effective community mitigation to reduce exposure to severe acute respiratory syndrome coronavirus 2 (SARS-CoV-2).

Our prior study was the first published study on the extent to which widely viewed YouTube videos address voluntary behaviors for effective community mitigation [2]. These videos, which were uploaded during January 2020, showed that fewer than one-third of the videos addressed any of the behaviors recommended by the US CDC to protect oneself and others by reducing exposure to SARS-CoV-2—the essence of effective community mitigation [3]. Because of the rapidly changing nature of YouTube in the context of the COVID-19 pandemic, we conducted a follow-up study to identify the most widely viewed YouTube videos as of March 20, 2020 to determine how coverage of preventive behaviors for effective community mitigation has changed.

## Methods

A successive sampling design was used to select the 100 most widely viewed YouTube videos on COVID-19 as of January 31, 2020, and March 20, 2020. In both cases, the methods described in our prior study [2] were followed. In the second sampling period, half of the videos were coded by author CHB and the other half were coded by author CJ. Interrater reliability was previously demonstrated and found to be excellent (Cohen kappa=0.97). The prior study focused on preventive behaviors, mortality and fear, symptoms, transmission and natural history, and other precautions, while this study focuses exclusively on prevention behaviors to mitigate community transmission. Data analysis involved descriptive statistics, and all analyses were conducted using SPSS, version 26 (IBM Corp).

## Results

At the time of data collection (March 20, 2020), the videos in the sample were viewed more than 355 million times (by the afternoon of April 5, 2020, these videos garnered almost 59 million additional views; total=413,975,717 views). The mean number of views per video was 3,552,125 (SD 2,817,911), and the mean length was 12.3 minutes (SD 11.3 minutes; range 34 seconds to 89 minutes). Most were created in English (n=79, 79.0%) or with English subtitles (n=1, 1.0%), and 20.0% were in Spanish. The large majority (n=95, 95.0%) featured a live presenter, while 5.0% (n=5) featured animation.

Although all the videos in the prior study were uploaded by three sources—consumers (11%), healthcare professionals (4%), and news (85%)—by March 20, 2020, the majority (57%) of the most widely viewed videos in this study were uploaded by entertainment television, garnering almost 55% (n=193,639,691) of the total cumulative views (Tables 1 and 2). There was a large decline in the number of videos uploaded by news sources (from 85 to 19) and a commensurate decline in the proportion of cumulative views amassed from this source (from 82% to 22.5%). In contrast, there was an increase in the number of videos uploaded by consumers from 11 to 19, with the proportion of cumulative views changing from 13.8% to 18.7%. The number of videos and the proportion of cumulative views garnered by videos uploaded by professionals remained essentially unchanged (4 versus 5 and 4.2% versus 4.3%, respectively). In the short time between our first and second successive samples (48 days), there was a dramatic increase in cumulative views garnered by the 100 most widely YouTube videos (from 125,286,561 to 355,212,487). It is noteworthy that only 5 of the videos from the first sample were retained in the second sample.

Fewer than half of the videos covered any of the 8 prevention behaviors recommended by the US CDC as of March 2020. In January, 39 videos garnering almost 60 million views covered the topic of staying indoors, while in March, this recommendation was covered in 42 videos garnering over 160 million views. There was a large increase in the number and proportion of cumulative views garnered by videos regarding hand hygiene, from 33,268,243 (26.6%) to 182,331,135 (51.3%). There were also increases in the number of videos and the proportion of cumulative views garnered regarding staying home when ill and covering cough/sneeze with tissue and discarding it in the trash. In contrast, there was a decline in number of videos addressing avoiding close contact with people who are ill (from 31 to 18), even though the number of views garnered by these videos increased (from 41,269,546 to 97,013,939). In the first sample, use of a facemask for protection if you are caring for someone who is ill was not mentioned and facemask use for protecting others if you are ill was only mentioned in 2 videos; in the second sample, the first topic was covered in 8 videos that were viewed over 26 million times and the second topic was covered in 4 videos viewed over 13 million times. Cleaning and disinfecting highly touched objects and surfaces was addressed in 16 videos in the first sample (with 17,545,061 views) and 15 videos in the second sample (with 70,365,530 views).

**Table 1:** Behaviors to mitigate transmission of COVID-19 covered in widely viewed YouTube videos by source in January 2020.

Prevention behaviors	Total		Consumer			Professional			News		
	Total number of views (%)^a^	Total number of videos		Number of views (%)^b^	Number of videos (%)^b^		Number of views (%)^b^	Number of videos (%)^b^		Number of views (%)^b^	Number of videos (%)^b^
Overall	125,286,561 (100)	100		17,288,306 (13.8)	11 (11.0)		5,299,489 (4.2)	4 (4.0)		102,698,766 (82)	85 (85.0)
Stay indoors^c^	59,527,347 (47.5)	39		7,333,961 (12.3)	6 (15.4)		3,800,508 (6.4)	2 (5.1)		48,392,878 (81.3)	31 (79.5)
Hand hygiene	33,268,243 (26.6)	26		4,869,024 (14.6)	4 (15.4)		3,910,326 (11.8)	2 (7.7)		24,488,893 (73.6)	20 (76.9)
Avoid close contact with those who are sick	41,269,546 (32.9)	31		7,409,099 (18.0)	5 (16.1)		4,734,854 (11.5)	3 (9.7)		29,125,593 (70.6)	23 (74.2)
Stay home when ill	42,647,990 (34.0)	29		7,409,099 (17.4)	5 (17.2)		4,060,401 (9.5)	2 (6.9)		31,178,490 (73.1)	22 (75.9)
Cover cough/sneeze with tissue; throw tissue away	19,625,830 (15.7)	14		4,150,801 (21.1)	3 (21.4)		3,235,873 (16.5)	1 (7.1)		12,239,156 (62.4)	10 (71.4)
Use facemask for protection if you are caring for the ill	0 (0)	0		0 (0)	0 (0)		0 (0)	0 (0)		0 (0)	0 (0)
Use facemask for protecting others if you are ill	1,152,765 (0.9)	2		627,664 (54.4)	1 (50.0)		0 (0.0)	0 (0.0)		525,101 (45.6)	1 (50.0)
Clean and disinfect highly touched objects and surfaces	17,545,061 (14.0)	16		4,869,024 (27.8)	4 (25.0)		3,910,326 (22.2)	2 (12.5)		8,765,711 (50.0)	10 (62.5)

^a^Column percentage.

^b^Row percentage.

^c^Universal recommendation to stay indoors did not exist in January but was coded under Other Precautions.

**Table 2:** Behaviors to mitigate transmission of COVID-19 covered in widely viewed YouTube videos by source in March 2020.

Prevention behaviors	Total		Consumer		Professional		News		Entertainment	
	Total number of views (%)^a^	Total number of videos	Number of views (%)^b^	Number of videos (%)^b^	Number of views (%)^b^	Number of videos (%)^b^	Number of views (%)^b^	Number of videos (%)^b^	Number of views (%)^b^	Number of videos (%)^b^
Overall	355,212,487 (100)	100	66,303,762 (18.7)	19 (19.0)	15,213,523 (4.3)	5 (5.0)	80,055,511 (22.5)	19 (19.0)	193,639,691 (54.5)	57 (57.0)
Stay indoors^c^	160,105,457 (45.1)	42	25,633,078 (16.0)	6 (14.3)	2,147,978 (1.3)	1 (2.4)	33,653,431 (21.0)	7 (16.7)	98,670,970 (61.6)	28 (66.7)
Hand hygiene	182,331,135 (51.3)	44	50,255,990 (27.6)	11 (25.0)	2,147,978 (1.2)	1 (2.3)	56,645,499 (31.1)	12 (27.3)	73,281,668 (40.2)	20 (45.5)
Avoid close contact with those who are sick	97,013,939 (27.3)	18	31,374,132 (32.3)	4 (22.2)	2,147,978 (2.2)	1 (5.6)	16,835,274 (17.4)	3 (16.7)	46,656,555 (48.1)	10 (55.6)
Stay home when ill	163,220,603 (46.0)	44	47,505,715 (29.1)	10 (22.7)	2,147,978 (1.3)	1 (2.3)	35,502,777 (21.8)	7 (15.9)	78,064,133 (47.8)	26 (59.1)
Cover cough/sneeze with tissue; throw tissue away	98,060,105 (27.6)	24	31,620,076 (32.2)	8 (33.3)	2,147,978 (2.2)	1 (4.2)	31,260,481 (31.9)	6 (25.0)	33,031,570 (33.7)	9 (37.5)
Use facemask for protection if you are caring for the ill	26,881,257 (7.6)	8	2,205,478 (8.2)	1 (12.5)	0 (0)	0 (0)	9,151,436 (34.0)	1 (12.5)	15,524,343 (57.8)	6 (75.0)
Use facemask for protecting others if you are ill	13,491,951 (3.8)	4	0 (0)	0 (0)	0 (0)	0 (0)	9,151,436 (67.8)	1 (25.0)	4,340,515 (32.2)	3 (75.0)
Clean and disinfect highly touched objects and surfaces	70,365,530 (19.8)	15	34,705,722 (49.3)	6 (40.0)	2147978 (3.1)	1 (6.7)	15,191,934 (21.6)	4 (26.7)	18,319,896 (26.0)	4 (26.7)

^a^Column percentage.

^b^Row percentage.

^c^Universal recommendation to stay indoors did not exist in January but was coded under Other Precautions.

## Discussion

Over 125 million views in our first sample and the dramatic increase to over 355 million views in our second sample demonstrates the incredible reach of YouTube for communicating and mobilizing the public about community mitigation as a means to reduce mortality from the COVID-19 viral pandemic. YouTube is one of the most effective means for increasing awareness and interest in community mitigation of COVID-19 not only because of its widespread reach but also because many vulnerable people within the population may have low levels of literacy, which makes reading and deciphering behavioral recommendations described on websites difficult or impossible. Our prior studies on emerging infectious diseases such as Zika [4], Ebola [5], and other public health problems affecting population health [6-8] further demonstrate the reach of YouTube as a way to help educate people and help them make informed decisions. However, there has never been a more urgent need for such education to mobilize and engage people in communities throughout the United States and globally to understand and practice behaviors to mitigate community transmission as with the COVID-19 viral pandemic.

A highlight of the findings is the dramatic change that occurred from the first to the second sample, not only in the number of cumulative views, but also in the sources of videos that were most likely to have a widespread reach. Although there were no videos uploaded by entertainment television in our first sample, within 7 weeks, this source comprised the majority of videos (57%) and garnered the majority of cumulative views (>193 million). At this critical time, as all sectors of the American public work together toward the goal of community mitigation, we believe our findings indicate the potential role of entertainment television in saving lives. The implication is that in addition to holding regular press briefings covered by national news, public health officials may be able to achieve our collective goal of community mitigation by appearing on entertainment television and communicating clearly about the specific behaviors that people must practice to protect themselves, their families, and their communities, especially the many health care professionals and essential workers placing themselves at risk to care for others.

The behaviors we studied were identified from the US CDC website [3], but we collapsed behaviors into categories that could have been delineated in greater detail. For example, the recommendation regarding “Clean your hands often,” which we entitled hand hygiene, includes very specific advice: “Wash your hands often with soap and water for at least 20 seconds especially after you have been in a public place, or after blowing your nose, coughing, or sneezing” [3]. This single recommendation can be disaggregated into 9 specific behaviors: Wash your hands (1) often (2) with soap (3) and water (4) for at least 20 seconds (5) especially after you have been in a public place (6), or after blowing your nose (7), coughing (8), or sneezing (9). In this same category of hand hygiene, additional recommendations pertain to the use of hand sanitizer containing at least 60% alcohol and covering all surfaces of hands and rubbing them together until they feel dry and to “avoid touching your eyes, nose, and mouth with unwashed hands” [3]. These recommendations too comprise many behaviors, which we did not specifically delineate in our coding protocol. We believe that the complexity of these behavioral recommendations highlights the value of video presentation in communicating about and demonstrating desired behaviors.

It should be noted that new knowledge, including recommended behaviors to mitigate community spread of COVID-19, are emerging rapidly and it is important to track the extent to which widely viewed videos cover up-to-date accurate information. Despite its great potential for disease prevention, it is important to identify and dispel inaccurate information that may be conveyed on YouTube, which we have documented in our previous studies on other topics [6,7]. Almost all published studies on YouTube and public health are cross-sectional, but we believe ongoing tracking of content contained in YouTube videos is necessary to improve understanding about the kinds of information people need to make informed decisions, which is especially urgent in the current virus pandemic.

Given that there is currently no vaccine to reduce personal susceptibility and no proven treatment therapies, educating and mobilizing people to practice the behaviors that we know will reduce exposure to SARS-CoV-2 is the best and only hope for dramatically reducing the number of lives that will be lost. We believe YouTube can play an important role in achieving that goal. Such communication should be a key element of a comprehensive national (and global) strategy for educating, mobilizing, and engaging the public to adopt and practice behaviors for community mitigation.

## Abbreviations

CDC: Centers for Disease Control and Prevention
COVID-19: coronavirus disease
SARS-CoV-2: severe acute respiratory syndrome coronavirus 2
